# A Case of Secondary Hemophagocytic Lymphohistiocytosis in a Patient With T-cell Lymphoma

**DOI:** 10.7759/cureus.56558

**Published:** 2024-03-20

**Authors:** Bishara Jahshan, Anna B Owczarczyk, Hamed Daw, Abdo Haddad

**Affiliations:** 1 Internal Medicine, Unity Hospital/Rochester Regional Health, Rochester, USA; 2 Pathology and Laboratory Medicine, Robert J. Tomsich Pathology and Laboratory Medicine Institute, Cleveland Clinic, Cleveland, USA; 3 Hematology and Oncology, Cleveland Clinic/Fairview Hospital, Cleveland, USA

**Keywords:** autoimmune, lymphoproliferative neoplasm, diagnosis, t-cell lymphoma, hemophagocytic lymphohistocytosis (hlh)

## Abstract

Hemophagocytic lymphohistiocytosis (HLH) is a rare and life-threatening condition that results from excessive immune activation and inflammation. This condition may be triggered by various factors, including infections, malignancies, or autoimmune diseases. Here, we report the case of a 39-year-old male who developed HLH secondary to T-cell lymphoma and had a history of multiple autoimmune disorders.

Our patient presented with shortness of breath and weakness which led to an admission for methicillin-resistant *Staphylococcus aureus* bacteremia. His hospital course deteriorated rapidly due to his worsening condition. He was confirmed to have HLH based on the HLH-2004 criteria with the presence of fever, splenomegaly, hypertriglyceridemia, hypofibrinogenemia, low natural killer cell function, high ferritin, and soluble interleukin 2 receptor levels. Peripheral blood smear and bone marrow biopsy showed atypical lymphocytes consistent with a T-cell lymphoma, but no hemophagocytosis. He was treated with dexamethasone and etoposide. Despite treatment, the patient passed away.

This case aims to contribute further to the understanding of secondary HLH in the setting of T-cell lymphoma. It also illuminates how vital early recognition and treatment are in patients with secondary HLH.

## Introduction

Hemophagocytic lymphohistiocytosis (HLH) is a rare, life-threatening hematological disorder characterized by intense inflammation and dysregulated immune activation [1]. Secondary HLH is commonly triggered by infections or malignancies but may also be induced by autoimmune disorders [2]. It has a poor prognosis, and if left untreated, survival ranges from mere days to weeks due to multiorgan failure, with overall mortality ranging from 41% to 75%. Early recognition and treatment are extremely important to limit the high morbidity and mortality of this disease [3]. While HLH is predominantly recognized in the pediatric population, it is imperative to acknowledge its occurrence in adults, where it frequently manifests as a secondary condition associated with underlying malignancies or immune dysregulation.

In this case, we present an adult patient who presented with secondary HLH in the setting of a T-cell lymphoma.

## Case presentation

We present a case involving a 39-year-old male patient with a past medical history of type 1 diabetes mellitus, autoimmune enteropathy, rheumatoid arthritis, hypogammaglobulinemia, and hypothyroidism who presented to the hospital for shortness of breath.

The patient presented with a two-day history of shortness of breath, accompanied by weakness. At presentation, the patient reported no fever, cough, sputum production, or recent sick contacts. Previously, he had undergone multiple hospitalizations for recurrent infections, notably parainfluenza pneumonia, requiring intensive care unit (ICU) admission and intubation.

The patient’s home medications included prednisone, leflunomide, insulin, and levothyroxine. He was a non-smoker. Additionally, he abstained from alcohol and intravenous drug use. He solely reported occasional marijuana use.

The physical examination revealed tachycardia with a heart rate of 102 beats per minute and hypoxia with an oxygen saturation of 85%, prompting the administration of 2 L of oxygen to maintain adequate oxygen saturation. The general appearance indicated mild distress, and mucous membranes appeared dry. On respiratory auscultation, rales were noted bilaterally in the lungs. No signs of lymphadenopathy were observed. There was no significant lower extremity swelling, redness, or tenderness.

Blood work indicated no elevation in his leukocyte count but revealed thrombocytopenia, with platelets at 60,000/μL (normal range: 150,000-450,000/μL). Electrolytes were within the normal range, and his kidney function was normal. D-dimer was elevated at 2,740 ng/mL (normal range: <500 ng/mL). A chest X-ray and a CT of the chest with intravenous contrast showed no pulmonary embolism but revealed resolving infiltrates from the previous parainfluenza infection. Abdominal ultrasound demonstrated no gallbladder disease but did reveal mild splenomegaly at 15 cm.

Eventually, the patient was admitted for the management of acute hypoxic respiratory failure secondary to pneumonia. Subsequently, he developed a fever of 38.5°C, and a blood culture collected on admission grew methicillin-resistant *Staphylococcus aureus* (MRSA).

Hematology was consulted for the thrombocytopenia. Diagnosis of HLH was suspected, and the patient received dexamethasone (10 mg/m² daily) and etoposide (150 mg/m² twice weekly) while undergoing further diagnostic workup. The patient exhibited a fever of 38.5°C, confirmed splenomegaly through ultrasound, hypertriglyceridemia (281 mg/dL), hypofibrinogenemia (99 mg/dL), natural killer cell function less than 1%, and elevated ferritin levels (1,906 ng/mL). Additionally, there was an elevation in the soluble interleukin 2 alpha receptor level, measuring 74,993 U/mL (see Table 1). These collective findings fulfilled the HLH-2004 diagnostic criteria (see Table 2) [4], confirming the diagnosis of HLH.

**Table 1:** Laboratory analysis. IL-2 = interleukin 2; NK = natural killer

Test	Result	Reference Range
Platelet count	60,000/μL	150,000–450,000/μL
Triglycerides	281 mg/dL	<150 mg/dL
Fibrinogen	99 mg/dL	200–400 mg/dL
NK cell function	<1%	Normal: >8%
Ferritin	1.906 ng/mL	Male: 20–500 ng/mL; Female: 10–150 ng/mL
Soluble IL-2 alpha receptor	74,993 U/mL	250–2,000 U/mL

**Table 2:** HLH diagnostic criteria. HLH = hemophagocytic lymphohistiocytosis; NK = natural killer

Criteria	Description
Fever	≥38.5°C
Splenomegaly	Enlargement of the spleen
Cytopenias	At least two of the following: Hemoglobin <9 g/dL, platelets <100,000/mm³, neutrophils <1,000/mm³
Hypertriglyceridemia	Fasting triglycerides ≥265 mg/dL
Hemophagocytosis	Presence of hemophagocytic histiocytes in bone marrow, spleen, or lymph nodes
Low or absent NK cell activity	Decreased or absent NK cell activity
Elevated ferritin	Ferritin ≥500 ng/mL
Soluble CD25	Soluble CD25 (sIL-2 receptor alpha) ≥2,400 U/mL

**Figure 1:**
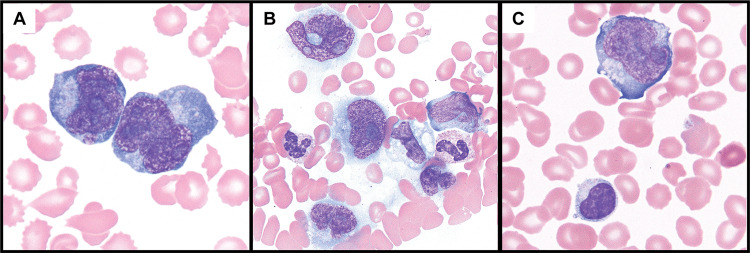
Peripheral blood smear. Circulating T-cell lymphoma cells on the peripheral blood smear. (A) The lymphoma cells are intermediate to large in size with markedly irregular nuclear contours, condensed chromatin, single to multiple prominent nucleoli, and basophilic cytoplasm (100×). (B) The lymphoma cells are larger than normal circulating neutrophils and monocytes (60×). (C) Lymphoma cell (top) compared to a reactive, non-neoplastic lymphocyte (100×).

**Figure 2:**
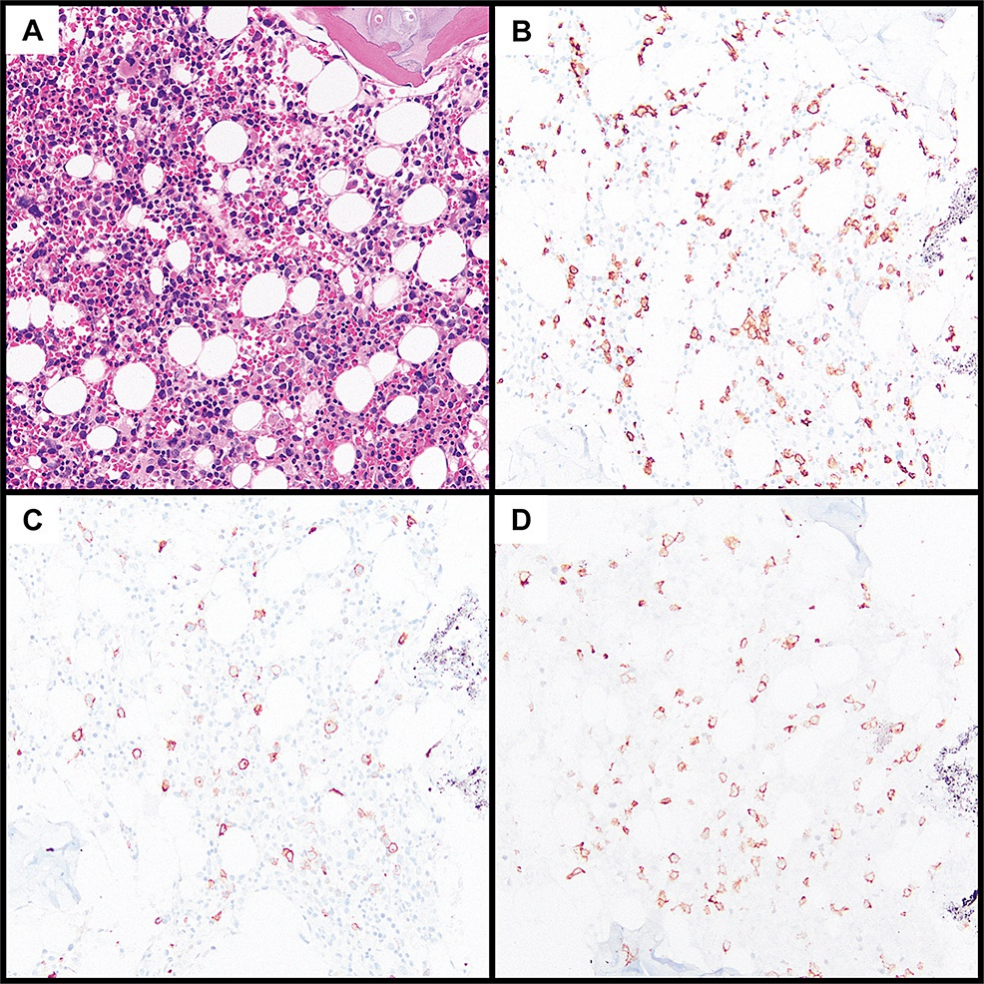
Bone marrow biopsy. T-cell lymphoma involving the bone marrow. (A) Hematoxylin and eosin-stained section from the bone marrow core biopsy, showing a representative area of trilineage hematopoiesis with scattered atypical lymphocytes. These lymphocytes are best appreciated by immunohistochemistry, positive for CD3 (B), CD30 (C, subset), and TCR delta (D). All images captured at 20×.

Peripheral blood smear evaluation demonstrated circulating atypical lymphocytes compatible with lymphoma cells (Figure 1). Bone marrow analysis confirmed involvement by a T-cell lymphoma (approximately 10% of marrow cellularity, (Figure 2)), but showed no evidence of increased histiocytes or hemophagocytosis. On flow cytometry, these lymphoma cells were positive for CD3 (surface and cytoplasmic) and CD45 and negative for CD34, TdT, and all other tested T-cell markers, including TRBC1, CD2, CD4, CD5, CD7, CD8, and CD56. Further subclassification with additional molecular testing was not pursued.

The hospital course was complicated by shock, acute kidney injury, and encephalopathy, necessitating ICU admission for vasopressors, intubation, and continuous renal replacement therapy. Given his deterioration, the family changed his code status to comfort care. The patient passed away seven days after being admitted to the hospital.

## Discussion

It is estimated that around 50% of secondary HLH cases in North America and Europe are due to malignancy, while the other half of the cases are associated with rheumatological diseases or infections [5]. HLH has been reported in association with T-cell lymphoma in multiple instances [6-9] (see Table 3). Despite treatment, death was reported in those cases. What sets this case apart is the simultaneous presence of the most common predisposing factors to develop HLH, namely, autoimmune disease, infection, and malignancy.

**Table 3:** Reported cases of HLH secondary to T-cell lymphoma. HLH = hemophagocytic lymphohistiocytosis

Author:	Age (years)	Gender (male/female)	T-cell lymphoma type	Etoposide treatment (yes/no)	Outcome:
Khadanga et al. 2014 [6]	78	Female	T-cell non-Hodgkin’s lymphoma (CD30+ with aberrant T-cell phenotype)	No	Deceased
Paluszkiewicz et al. 2021 [7]	28	Male	Peripheral T-cell lymphoma not otherwise specified	Yes	Deceased
Lau et al. 2022 [8]	68	Female	Anaplastic large-cell lymphoma	No	Deceased
Kilani et al. 2022 [9]	48	Male	Not reported	Yes	Deceased

Despite not undergoing further subclassification with additional molecular and cytogenetic studies, the patient’s T-cell lymphoma falls under the category of iatrogenic immune-deficiency-associated lymphoproliferative disorder. Determining the instigator in the development of HLH in this case is difficult. In our opinion, the patient’s rheumatoid arthritis was an unlikely trigger of HLH, as it was well controlled with immunosuppressants. Thus, we favor the T-cell lymphoma as the likely culprit of the patient’s HLH, with the preceding infection acting as a catalyst. Unfortunately, regardless of the underlying cause, and despite the prompt diagnosis and treatment, the disease course was fatal.

## Conclusions

This case shows how autoimmune conditions, malignancies, and infections can trigger HLH. It underscores the importance of vigilant monitoring for the development of a lymphoproliferative disorder in patients on immunosuppression, which may manifest clinically as HLH. Furthermore, it emphasizes the necessity of a comprehensive and multidisciplinary approach in such cases. Ultimately, this case serves as a reminder of the potential lethality of HLH and the imperative for prompt and decisive therapeutic intervention to improve patient outcomes.
